# Central nervous system recurrence of diffuse large B-cell lymphoma – risk stratification and prophylaxis

**DOI:** 10.1016/j.htct.2026.106537

**Published:** 2026-09-18

**Authors:** Thamiris Silva Soares, Daniel Dias Ribeiro

**Affiliations:** Universidade Federal de Minas Gerais (UFMG), Belo Horizonte, MG, Brazil

**Keywords:** Diffuse large B-cell lymphoma, CNS relapse, CNS prophylaxis, Secondary CNS involvement, Risk stratification

## Abstract

Diffuse large B-cell lymphoma is the most common subtype of non-Hodgkin lymphoma. Central nervous system relapse is an uncommon but devastating complication, associated with poor prognosis and limited therapeutic options. Over the years, multiple strategies have been investigated to reduce the risk of central nervous system recurrence, although their efficacy remains controversial.

This review aims to describe the evolution of central nervous system prophylaxis strategies in diffuse large B-cell lymphoma, highlighting risk stratification models, clinical outcomes, and emerging approaches designed to prevent central nervous system involvement.

## Introduction

Central nervous system (CNS) relapse of diffuse large B-cell lymphoma (DLBCL) not otherwise specified occurs in approximately 5–10% of patients during or after the end of treatment and presents unsatisfactory outcomes [1,2].

Although other lymphoma subtypes present a higher incidence of secondary CNS involvement and routinely adopt prophylactic strategies, the poor prognosis associated with CNS relapse in DLBCL has driven efforts to minimize this risk. Several studies have been carried out with the aim of stratifying patients as to their risk of relapse and evaluating prophylactic methods that would reduce these rates.

In the rituximab era, the German High-Grade Non-Hodgkin Lymphoma Study Group (DSHNHL) developed the Central Nervous System–International Prognostic Index (CNS-IPI) to stratify the risk of CNS relapse in patients with DLBCL treated with R-CHOP (Table 1) (rituximab, cyclophosphamide, doxorubicin, vincristine and prednisone). The model was derived from a cohort of over 2000 patients and incorporates one point for each conventional International Prognostic Index (IPI) factor (age >60 years, elevated lactate dehydrogenase [LDH], Eastern Cooperative Oncology Group [ECOG] performance status ≥2, Ann Arbor stage III–IV, and involvement of more than one extranodal site) along with renal or adrenal involvement or both. Patients classified as high risk (CNS-IPI ≥4) have an estimated CNS relapse risk of 9–10%, compared with approximately 5% and 1% in the intermediate- and low-risk groups, respectively. Although multiple extranodal sites were associated with CNS relapse in univariate analyses, only kidney, adrenal and skin involvement remained independently predictive after adjustment for IPI factors [3].

**Table 1:** Risk of CNS relapse according to the CNS-IPI.

Variable	
≥ 60 years	1 point
High LDH	1 point
ECOG performance status ≥2	1 point
Stage III-IV	1 point
Extranodal sites ≥ 2	1 point
Kidney/adrenal involvement	1 point
**CNS relapse risk classification**	**Risk of CNS relapse**
Low risk 0–1 point	1%
Intermediate risk 2–3 points	5%
High risk ≥ 4 points	9–10%

LDH: lactate dehydrogenase; ECOG: Eastern Cooperative Oncology Group; CNS: Central nervous system.

Other high-risk features not incorporated into the CNS-IPI include involvement of specific extranodal sites, such as the testes, breast, bone marrow, pericardium, and paranasal sinuses. Importantly, so-called double-hit lymphomas, defined by concurrent *MYC, BCL2* or *BCL6* rearrangements detected by fluorescence *in situ* hybridization (FISH), represent a biologically distinct high-risk entity and are associated with a substantially increased risk of CNS relapse. In contrast, double-expressor lymphomas, characterized by MYC and BCL2 protein overexpression on immunohistochemistry in the absence of underlying gene rearrangements, have also been associated with an elevated risk of CNS relapse. A study from the British Columbia Cancer Agency demonstrated higher two-year CNS relapse rates in double-expressor lymphomas compared with non-expressors (9.7% vs. 2.2%), as well as in cases of the activated B-cell–like (ABC) subtype according to the cell-of-origin classification using the Hans algorithm (9.1% vs. 2.1% for the germinal center B-cell–like [GCB] subtype). Additionally, tumors harboring high-frequency *MYD88* L265P or *CD79B* mutations, classified as the MCD genetic subtype and frequently observed in primary extranodal lymphomas involving immune-privileged sites such as the CNS, testis, and breast, have been associated with a markedly increased risk of CNS relapse (38% vs. 8%). Collectively, these data suggest that reliance on the CNS-IPI alone may be insufficient to accurately identify truly low-risk patients, particularly in the presence of adverse molecular features or high-risk extranodal disease involvement [4, 5, 6, 7, 8, 9].

Prophylactic strategies range from chemotherapy intensification to the administration of methotrexate, delivered either intrathecally (IT-MTX) or systemically at high doses (HD-MTX), with or without combining with other chemotherapeutic agents [8]. Table 2 summarizes the key studies evaluating strategies aimed at reducing the risk of CNS relapse.

**Table 2:** Studies demonstrating prophylactic methods of relapse in the central nervous system of patients with diffuse large B-cell lymphoma and their respective results.

	Study	Study characteristics	Age (years)	n	Chemotherapy	Criteria prophylaxis	Prophylaxis	CNS relapse Incidence	Pattern of CNS involvement
Rituximab	Boehme et al., 2009	Post hoc analysis of a randomized study	61–80	1222	(1) R-CHOP-14 (2) CHOP-14	MO, testicle, paranasal sinuses, orbit, oral cavity, tongue, salivary gland	IT-MTX 39%	(1) 4.1% (2) 6.9%	
	Ghose et al., 2015	Systematic review	> 60	4859	(1) R-CHOP/R-CHOEP (2) CHOP	Ranged from none, ≥ 1 risk factor to all patients	Rituximab, IT-MTX	(1) 5.52% ± 2.21% (2) 4.43% ± 0.9% P < 0.05	Leptomeningeal involvement was less frequent in the rituximab group (≈16% vs. 39%; p < 0.001)
	Villa et al., .2009	Retrospective	≥ 16	435	(1) R-CHOP (2) CHOP	Stages III/IV or I/II with B symptoms and/or Bulky disease, testicular lymphoma	IT-MTX	7.1%	Predominance of isolated parenchymal CNS relapses in rituximab-treated patients (63%vs. 25%).
Intensification of chemotherapy	Mcmillian et al., 2020	Prospective phase II	18–65	111	R-CODOX-M-IVAC		IT-MTX and HD-MTX for everyone as part of the protocol	3.6%	
	Holte et al., 2013	Prospective phase II	18–65	145	R-CHOEP-14		Cytarabine and HD-MTX	4.5%	
	Tilly et al., 2003	Prospective phase II	< 70	974	(1)ACVBP (2)CHOP		(1) IT-MTX, HD-MTX and consolidation (ifosfamide, etoposide and cytarabine)	(1) 2.8% (2) 8.3%	
	Thieblemont et al., 2021	Prospective phase II	18–60	2203	(1) R-ACVBP followed by consolidation depending on the risk with HD-MTX or HD-MTX+ASCT (2) R-CHOP/R-CHOEP/RmegaCHOEP		(1) HD-MTX or (2) IT-MTX	HD-MTX+ASCT	(1) 0% (2) 1% p 0.5
IT prophylaxis	Eyre et al., 2020	Systematic review		7357	R-CHOP/CHOP/ DA-EPOCH-R		IT 11,9%	1.9 a 8.4%	
HD-MTX prophylaxis	Wilson et al., 2022	Retrospective	≥ 16	1384	R-CHOP or MACOP-B±R	Without specific criteria, all received HD-MTX	(1) HD-MTX interspersed with chemoimmunotherapy (2) HD-MTX end of treatment	(1) 5.7% (2) 5.8%	53% parenchyma, 21% parenchyma and leptomeningeal and 27% leptomeningeal
	Cheah et al., 2014	Retrospective	16–84	(1) 49 (2) 125 (3) 43	(1) R-CHOP + IT-MTX (2) R-CHOP + HD-MTX (3) RHCVAD or RCODOX-M-IVAC	≥2 of the following: multiple extranodal sites, elevated LDH, B symptoms; or extra-nodal involvement of any of the following: bone marrow, breast, testicles, kidney, adrenal, paranasal sinuses, nasopharynx, liver, paravertebral	(1) IT-MTX (2) HD-MTX x 2 (3) IT or HD-MTX as part of the protocol	(1) 18.4% (2) 6.9% (3) 2.3%	
	Lewis et al., 2022	Retrospective	18–80	2267	R-CHOP/ DA-EPOCH-R	High-risk IPI-CNS, double hit, primary breast and testicular lymphoma	(1) No prophylaxis (2) HD-MTX	(1) 8.4% (2) 9.1%	
IT and HD-MTX prophylaxis	Orellana-Noia et al., 2022	Retrospective	> 18	1162	R-CHOP/EPOCH-R		(1)20.9% HD-MTX (2) 79.1% IT-MTX	(1) 6.8% (2) 5.4% p 0.4	

DLBCL, diffuse large B-cell lymphoma; CNS, central nervous system; MO, bone marrow; IPI, International Prognostic Index; R-CHOP, Rituximab, cyclophosphamide, doxorubicin, vincristine and prednisone; DA-EPOCH-R, Rituximab, etoposide, doxorubicin, vincristine, prednisone, cyclophosphamide; R-CODOX-M-IVAC (Rituximab, cyclophosphamide, vincristine, doxorubicin, methotrexate, ifosfamide, etoposide and cytarabine); R-CHOEP-14 (R-CHOP associated with etoposide) followed by cytarabine and high-dose methotrexate; ASCT, autologous stem cell transplant; ACVBP (doxorubicin, cyclophosphamide, vindesine, bleomycin, prednisone) followed by intrathecal methotrexate, two cycles of high-dose methotrexate of 3 g/m² and consolidation with four cycles of ifosfamide–etoposide and cytarabine in some patients; IT-MTX, intrathecal methotrexate; HD-MTX, high-dose methotrexate.

Since the introduction of rituximab, the prognosis of DLBCL has improved considerably, and this benefit has been hypothesized to extend to a reduced risk of CNS relapse. Intravenous rituximab is a large monoclonal antibody that does not cross the blood–brain barrier and therefore does not achieve therapeutic concentrations in the cerebrospinal fluid (CSF) or brain parenchyma. Nevertheless, it may exert indirect cytotoxic effects by improving systemic disease control, thereby potentially reducing the risk of secondary CNS involvement [10]. In the RICOVER-60 trial, which randomized 1222 patients to receive R-CHOP-14 or CHOP-14, CNS relapse occurred in 58 patients (4.8%); with a higher incidence in the CHOP-14 group compared with the R-CHOP-14 group (6.9% vs. 4.1%, respectively; p = 0.043), suggesting a protective effect of rituximab against CNS relapse. However, Ghose et al., in a systematic review including seven studies, among them RICOVER-60, compared R-CHOP/R-CHOEP with CHOP and reported conflicting results. The mean incidence of CNS relapse was 5.52% ± 2.21% in the rituximab-containing regimens versus 4.43% ± 0.9% in the CHOP group (p < 0.05). Notably, they observed a higher proportion of leptomeningeal relapses among patients treated with rituximab. In contrast, Villa et al., in an analysis of 435 patients, reported a higher proportion of parenchymal CNS relapses compared with leptomeningeal or combined leptomeningeal/parenchymal involvement in patients treated with rituximab (63%vs. 25%). This pattern is consistent with the limited penetration of rituximab into the CNS parenchyma [3,11, 12, 13, 14, 15].

Given these conflicting data regarding the protective role of rituximab against CNS relapse, subsequent studies evaluated whether intensification of chemoimmunotherapy with CNS-penetrating agents could serve as an alternative prophylactic strategy in aggressive B-cell lymphomas. Overall, these approaches did not demonstrate a substantial impact on CNS relapse rates. It has been hypothesized that more intensive regimens may reduce CNS relapse either by achieving superior systemic disease control or by incorporating agents with intrinsic CNS penetration. Puckrin et al. reported lower CNS relapse rates among high-risk patients treated with intensive frontline chemoimmunotherapy or consolidative autologous stem cell transplantation, suggesting that optimization of first-line systemic therapy may represent a more effective strategy to mitigate CNS recurrence risk [16].

In an effort to further reduce secondary CNS involvement, IT-MTX emerged as the earliest prophylactic strategy. However, most studies evaluating intrathecal prophylaxis are challenging to interpret due to their retrospective or post hoc design, heterogeneity in patient selection, variability in chemotherapy regimens and dosing schedules, concomitant use of high-dose systemic antimetabolites, and the overall low incidence of CNS relapse events. A systematic review conducted by Eyre et al. in the rituximab era assessed the role of intrathecal prophylaxis across three post hoc analyses (RICOVER-60, R-CHOP-14/21, and GOYA), one prospective database, and ten retrospective series, encompassing a total of 7357 patients. A median of 11.9% of patients received intrathecal prophylaxis, irrespective of risk group or at the investigator’s discretion. The cumulative incidence of CNS relapse ranged from 1.9% at 6.5 years to 8.4% at five years, with the majority of relapses (73%) involving the brain parenchyma. In the RICOVER-60 study specifically, patients considered at high risk, defined by bone marrow or testicular involvement or disease involving the upper neck or head, received IT-MTX. In a multivariable Cox regression model adjusting for IPI factors, a significant interaction was observed between IT-MTX exposure and rituximab use (relative risk: 6.1). CNS relapse risk was significantly reduced with IT-MTX in patients treated with CHOP alone, whereas no benefit was observed in the R-CHOP group. Importantly, these findings are derived largely from historical cohorts treated without rituximab and should not be extrapolated to contemporary clinical practice, in which rituximab-based immunochemotherapy is standard of care 5, 8, 14.

IT-MTX, often combined with cytarabine or corticosteroids or both, has historically been the most commonly used strategy for CNS prophylaxis in oncology. However, since 2009, its efficacy has been increasingly questioned. Accumulating evidence indicates that IT-MTX does not effectively prevent CNS relapse in the rituximab era, prompting the investigation of alternative strategies, particularly the use of systemic high-dose chemotherapy capable of penetrating the CNS, such as HD-MTX [5,8,14,17, 18, 19].

Given that CNS relapses in the rituximab era predominantly involve the brain parenchyma, HD-MTX emerged as a potentially more effective prophylactic approach for patients with high-risk DLBCL, mirroring its established role in the treatment of primary CNS lymphomas [17, 18, 19, 20].

In a multicenter retrospective study, Cheah et al. evaluated the role of HD-MTX in preventing CNS relapse among patients with DLBCL. A total of 217 patients were analyzed and stratified into three groups: (1) R-CHOP or MACOP-B ± rituximab, in which methotrexate doses were below 1 g/m² and therefore unlikely to achieve therapeutic concentrations in the brain parenchyma; (2) R-CHOP–like regimens combined with HD-MTX; and (3) Hyper-CVAD or R-CODOX-M/IVAC, both of which incorporate HD-MTX. The three-year CNS relapse rates were 18.4% (95% CI: 9.5–33.1%), 6.9% (95% CI: 3.5–13.4%), and 2.3% (95% CI: 0.4–15.4%) in Groups 1, 2, and 3, respectively (p = 0.009). Given the markedly higher CNS relapse rate in the group that did not receive HD-MTX, these findings suggest a potential protective effect of systemic HD-MTX [21].

A large retrospective cohort study evaluated 2300 patients treated in the rituximab era who were considered at high risk for CNS relapse, defined by a CNS-IPI score of 4–6 (89.2%), high-grade B-cell lymphoma with *MYC* rearrangements associated or not with *BCL2* or *BCL6* or both rearrangements, primary testicular lymphoma, or primary breast DLBCL. Most patients (93.8%) received R-CHOP–like regimens, and outcomes were analyzed according to the use of HD-MTX for CNS prophylaxis. Overall, 410 patients (17.8%) received HD-MTX. CNS relapse occurred in 32 of 410 patients (7.8%) in the HD-MTX group, compared with 169 of 1890 patients (8.9%) who did not receive HD-MTX. The adjusted five-year cumulative incidence of CNS relapse was 8.4% in the HD-MTX group versus 9.1% in the non–HD-MTX group (p = 0.10). Among patients who achieved a complete response, 284 of 1455 (19.5%) received HD-MTX, of whom 16 (5.6%) experienced CNS relapse, compared with 68 of 1171 patients (5.8%) who did not receive HD-MTX. No significant difference in the five-year risk of CNS relapse was observed between the groups (5.0% vs. 6.0%). The authors estimated that, assuming a true absolute risk reduction of 1% in favor of HD-MTX, approximately 100 patients would need to be treated to prevent a single CNS relapse [5].

Although early retrospective series suggested a potential protective effect of HD-MTX, several large contemporary retrospective studies have failed to demonstrate a meaningful reduction in CNS relapse rates, which remain in the range of 6–12% among patients with high-risk aggressive B-cell lymphoma. Interpretation of these data requires caution, given the heterogeneity in risk stratification across studies and the preferential use of HD-MTX in younger and fitter patients, introducing substantial selection bias [19].

Finally, a recent multicenter study directly compared IT-MTX with HD-MTX for CNS prophylaxis. Orellana-Noia et al. analyzed outcomes from 1162 patients treated at 21 U.S. academic institutions who received CNS prophylaxis (IT-MTX: n = 894; HD-MTX: n = 236). According to CNS-IPI risk categories, CNS relapse rates were 5.1% in low-risk, 3.8% in intermediate-risk, and 8.4% in high-risk patients (p = 0.02). After excluding patients with testicular involvement from CNS-IPI stratification, adjusted CNS relapse rates were 3.6%, 3.7%, and 8.2% for low-, intermediate-, and high-risk groups, respectively, with an overall rate of 5.0%. CNS relapse rates did not differ significantly according to frontline chemoimmunotherapy regimen (R-CHOP vs. DA-EPOCH-R; OR = 1.25; 95% CI: 0.51–3.10; p = 0.74), even after adjustment for double-hit status and CNS-IPI. Similarly, no significant difference was observed according to the route of prophylaxis: 48 events (5.4%) in the IT-MTX group versus 16 events (6.8%) in the HD-MTX group (OR = 1.28; 95% CI: 0.71–2.30; p = 0.40). These findings were consistent across stratifications by CNS-IPI, NCCN-IPI, and double-hit status [8,19].

Given that CNS relapse typically occurs early, with a median time of approximately five months, an alternative hypothesis to consider is the presence of CNS involvement prior to the initiation of systemic therapy. The true frequency of asymptomatic CNS involvement at the time of DLBCL diagnosis remains unknown, as CSF evaluation and dedicated CNS imaging, including magnetic resonance imaging (MRI), are not routinely performed at baseline. Siegal & Goldschmidt postulated that CNS relapse in a subset of patients with DLBCL may result from occult lymphoma cells already present in the CNS at diagnosis, whereas in others it may arise from subsequent CNS dissemination. Hegde et al. evaluated 92 patients with newly diagnosed B-cell lymphomas, including DLBCL and Burkitt lymphoma, considered high risk for CNS involvement. Among the 51 patients who underwent CSF analysis, occult CNS disease, defined by positive flow cytometry despite negative cytology, was identified at diagnosis in 11 patients, five of whom subsequently developed CNS relapse. Although this cohort included patients with Burkitt lymphoma, a disease known to carry a particularly high risk of CNS involvement, these findings suggest that occult CNS disease at diagnosis cannot be disregarded in DLBCL. Furthermore, with the introduction of rituximab and the resulting predominance of parenchymal rather than leptomeningeal CNS relapse, reliance on CSF assessment alone likely underestimates the true burden of subclinical CNS involvement. This raises the possibility that a greater proportion of patients may harbor occult CNS disease at diagnosis, which could be detected through systematic baseline CNS imaging. Accordingly, an important first step in preventing secondary CNS involvement may be the systematic screening of patients at high risk, incorporating both CNS imaging and CSF analysis using cytology and flow cytometry. In parallel, the development and validation of more sensitive diagnostic tools, such as the detection of circulating tumor DNA in the CSF, may further improve the identification of minimal CNS disease at diagnosis [5,22,23].

Future strategies aimed at reducing CNS relapse in DLBCL are likely to rely less on intensification of conventional cytotoxic chemotherapy and more on novel agents with demonstrated CNS penetration. Immunomodulatory drugs such as lenalidomide and Bruton tyrosine kinase inhibitors, including ibrutinib, have shown clinical activity in CNS lymphoma, although results remain inconsistent. Notably, the phase III ROBUST trial and the phase II ECOG-ACRIN E1412 study, both comparing R-CHOP plus lenalidomide with R-CHOP alone, have not yet reported CNS-specific relapse outcomes. Similarly, the phase III PHOENIX trial comparing R-CHOP plus ibrutinib with R-CHOP in patients with non–germinal center DLBCL demonstrated low and comparable CNS relapse rates between treatment arms (2.4% vs. 3.8%). Finally, chimeric antigen receptor T-cell (CAR-T) therapy, an emerging option for relapsed or refractory DLBCL, has demonstrated meaningful activity in secondary CNS lymphoma, with responses observed in approximately half of treated patients. In the phase II ZUMA-12 trial, none of the 40 patients with DLBCL, a CNS-IPI score ≥3, and persistent positron emission tomography (PET) positivity after two cycles of anthracycline-based chemotherapy experienced CNS relapse following treatment with axicabtagene ciloleucel at a median follow-up of 15.9 months. Although preliminary, these findings suggest that early integration of CAR-T therapy may represent a promising strategy for CNS relapse prevention and warrant further investigation [17].

Despite the large number of studies conducted to date, most of which were retrospective, the benefit of CNS prophylaxis in DLBCL and the identification of the most effective strategy remain uncertain. Given the inconsistencies across the literature, the methodological limitations inherent to retrospective analyses, and the relative rarity of CNS relapse as an outcome, data interpretation and the development of evidence-based recommendations for risk stratification and prophylactic or therapeutic approaches remain challenging. Nonetheless, considering the non-negligible incidence of CNS relapse and its associated poor prognosis, continued risk assessment for CNS involvement remains warranted. Baseline CNS evaluation before treatment initiation, particularly in patients at high risk, represents a reasonable approach to identify occult disease and may inform subsequent management decisions. Until robust prospective randomized data become available and refinements in CNS risk classification are established, decisions regarding CNS prophylaxis and intensification of systemic therapy should be individualized and guided by clinical judgment. Ongoing and future studies exploring novel agents with CNS activity and alternative therapeutic strategies are essential to improve outcomes and to better define the optimal approach for preventing CNS relapse in DLBCL.

## Conflicts of interest

None.

## Data Availability

The data that support the findings of this study are available from the corresponding author upon reasonable request.
